# GPS/DR Error Estimation for Autonomous Vehicle Localization

**DOI:** 10.3390/s150820779

**Published:** 2015-08-21

**Authors:** Byung-Hyun Lee, Jong-Hwa Song, Jun-Hyuck Im, Sung-Hyuck Im, Moon-Beom Heo, Gyu-In Jee

**Affiliations:** 1 Electronics Engineering, Konkuk University, Seoul 143-701, Korea; E-Mails: maximan@konkuk.ac.kr (B.-H.L.); hwaya@konkuk.ac.kr (J.-H.S.); junhyuck@konkuk.ac.kr (J.-H.I.); 2 Satellite Navigation Team, Korea Aerospace Research Institute (KARI), Daejeon 305-806, Korea; E-Mails: ish@kari.re.kr (S.-H.I.); hmb@kari.re.kr (M.-B.H.)

**Keywords:** GPS/DR error estimation, lane level positioning, autonomous vehicles, vision, waypoint

## Abstract

Autonomous vehicles require highly reliable navigation capabilities. For example, a lane-following method cannot be applied in an intersection without lanes, and since typical lane detection is performed using a straight-line model, errors can occur when the lateral distance is estimated in curved sections due to a model mismatch. Therefore, this paper proposes a localization method that uses GPS/DR error estimation based on a lane detection method with curved lane models, stop line detection, and curve matching in order to improve the performance during waypoint following procedures. The advantage of using the proposed method is that position information can be provided for autonomous driving through intersections, in sections with sharp curves, and in curved sections following a straight section. The proposed method was applied in autonomous vehicles at an experimental site to evaluate its performance, and the results indicate that the positioning achieved accuracy at the sub-meter level.

## 1. Introduction

Autonomous land vehicles require a level of accuracy that can enable lane decisions. In prior research, Lee [1] and Serrano [2] obtained position accuracy that enabled taking lane decisions by using a precise positioning algorithm based on RTK using GPS with an open sky. Specifically, it is important to develop guidance systems for autonomous vehicles since autonomous vehicles have to follow particular paths. Although autonomous driving is regionally possible by recognizing the surroundings, intelligent transportation services will only be commercially viable if the position of a vehicle is known with sufficient accuracy.

Therefore, the position information of vehicles should be sufficiently accurate to enable path- following functionality. If the information is sufficiently accurate and globally valid, then a safe path can be followed by implementing a simple guidance method. However, the GPS-RTK system requires data from a reference station and active communication links to receive all necessary information, so additional infrastructure is required. Also, standalone GPS with a Satellite Based Augmentation System (SBAS) cannot offer the necessary accuracy for the use of autonomous vehicles.

Waypoints and position information are necessary to guide vehicles, and inaccurate position information and waypoints can lead autonomous vehicles to wrong locations or to run in a biased route. Furthermore, lane keeping can be carried out using vision algorithms rather than position information. However, the disadvantage of using this method is that lanes must exist, so it cannot be used in areas where there is no guarantee of a continuity in lanes, such as in intersections and crosswalks. Moreover, problems can occur if lane detection is carried out in curved sections with straight lane models. Thus, the proposed method uses vision sensors and a curved line model to detect curved by calculating the lateral distance information of the detected lanes. In addition, stable autonomous driving methods have been suggested by estimating the GPS/DR errors through the use of longitudinal distance information by detecting stop lines and by matching the curved lane and the waypoints.

## 2. Related Work

Many studies have been carried out to localize vision-based and map-assisted methods [3,4,5,6], and computer vision and other techniques from robotic systems have been implemented to successfully follow paths without having absolute information on the position. Although such technologies have been previously applied in small areas, the DARPA Grand Challenge (DGC) in 2005 and DARPA Urban Challenge (DUC) in 2007 nevertheless required absolute position because the vehicle needed to navigate a large area.

Simultaneous Localization and Mapping (SLAM) is a popular mapping method (in this paper, a waypoint was used as a map). SLAM performs efficient localization with map building by using reflectivity information obtained with vision sensors, LiDAR, and an estimator, such as an Extended Kalman Filter (EKF) or a Rao-Blackwellized Particle Filter (RBPF) [7,8,9]. This is similar to the proposed method because SLAM performs localization through landmarks and a map. The difference is that SLAM uses all features for localization while the method proposed in this study performs localization via GPS/DR error estimation by detecting the lanes and stop lines.

Other methods use the intensity of 3D-LiDAR to generate surface maps of the road, and the data is then localized through map matching [10,11,12]. However, this cannot be applied in every vehicle because 3D-LiDAR is expensive and the surface map is a new data form that does not yet have a standard.

One of the localization method that uses vision sensor is visual odometry technique. Bak [13], Cuong [14], and Scaramuzza [15] used point features to extract odometry information of ego-vehicle. A lane, which is a line feature, is more apparent information than point features, and is used as a land mark in this paper.

Gruyer [16], Ieng [17] and Li [18] presented a localization method that uses lane detection that is similar to that proposed in this study in terms of the using lateral distance with mapping assistance. Especially, Gruyer and Ieng used lateral distance measurement using lateral camera systems which are placed outside of the vehicle. However, the proposed method performs lane tracking not in the image frame but in the vehicle frame by using lane tracking based on a curved model and reducing the lateral distance error that occurs when using the front camera as described in Section 4.2. The measurement equation that is derived in the lane frame is a difference with respect to other papers. In this manner, safe autonomous driving is supported in areas with a sharp curves by reducing the lateral distance error.

## 3. Localization Problems in Autonomous Driving

Autonomous vehicles should be able to perform continuous and reliable localization. The vehicle can be assumed to have a surveyed precise map that consists of waypoints that can be followed in addition to stop line information. The commands to follow the waypoints include the steering angle and speed. The position and the angle of the heading of vehicle are necessary in order to generate the proper steering angle and speed. A driving environment can be basically classified into three parts: a straight lane, a curved lane and a short region without a lane, such as an intersection. In a straight lane, it is possible to obtain the lateral distance by detecting the lane, and the navigation system accurately estimates the lateral position when the distance is measured. Also, the longitudinal position can be accurately estimated by using the distance measured when the stop line has been detected. Problems can occur at an intersection and in an area marking a transition from a straight to a curved section. Computer vision cannot be used to provide any information for the intersection, and no correction information is available for localization. In addition, the area that is to be converted into a curve from a straight lane has no longitudinal information, and the error of the longitudinal position results in steering commands that are either performed early or late with erroneous timing, which causes a departure from the lane. Therefore, autonomous driving with a precise lateral position is only possible in a straight section, but a precise longitudinal position is essential for autonomous vehicles in an area where a curved lane begins and were the vehicle will enter an intersection. In addition, the navigation system should provide the precise position at the intersection.

## 4. GPS/DR Error Estimator

**Figure 1:**
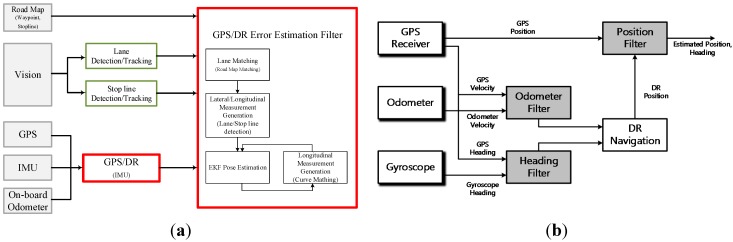
(**a**) Overall system structure; (**b**) GPS/DR system structure.

The proposed navigation system consists of a GPS, an Inertial Measurement Unit (IMU), an odometer, a map and a vision sensor (Figure 1a). The GPS/DR system consists of a GPS receiver, an odometer and a gyroscope (Figure 1b), and it provides the position and heading angle. The odometer filter estimates the velocity of the vehicle, the heading filter estimates the heading angle, and the position filter estimates the position and the heading of the GPS/DR system. The GPS/DR system model is summarized as follows.

Odometer filter:

(1) $$\delta SF_{odo}=w_{odo},z_{odo}=n\cdot \delta SF_{odo}+v_{odo}=V_{GPS}-V_{odo}$$

In this equation, $\delta SF_{odo}$ is the scale factor error of odometer, measurement $z_{odo}=V_{GPS}-V_{odo}$ is the difference between GPS and odometer speed and *n* is number of odometer pulses.

Heading filter:

(2) $$x_{head,k}=[\begin{array}{cc} 1 & \Delta t\\ 0 & 1 \end{array}][\begin{array}{c} \delta \theta \\ \delta B \end{array}]+w_{k-1}=F_{head,k-1}x_{head,k-1}+w_{k-1}$$

(3) $$z_{head,k}^{stop}=\left[\begin{array}{cc} 1 & 0 \end{array}\right]\left[\begin{array}{c} \delta \theta_{k}\\ \delta B_{k} \end{array}\right]+v_{k},z_{head,k}^{move}=\left[\begin{array}{cc} 0 & 1 \end{array}\right]\left[\begin{array}{c} \delta \theta_{k}\\ \delta B_{k} \end{array}\right]+v_{k}$$

In this equation, δθ and δB represent the heading angle error of the vehicle and the bias error of the gyroscope, $z_{head}^{move}$ is the measurement when the vehicle moves, and $z_{head}^{stop}$ is the measurement when the vehicle stopped.

**Figure 2:**
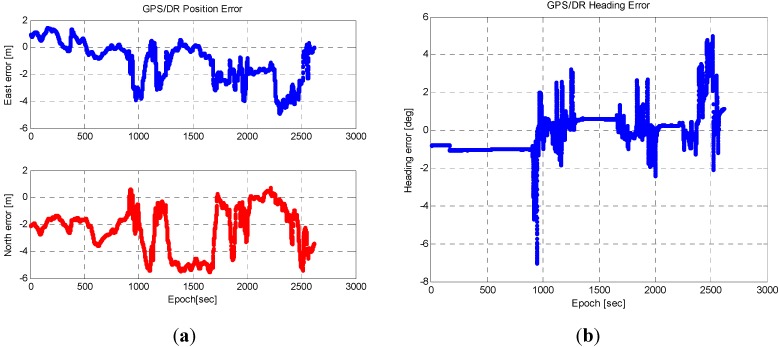
(**a**) GPS/DR position error; (**b**) GPS/DR heading error.

Figure 2 shows the performance of the estimated position/velocity of the GPS/DR system.

Image processing is used with a vision sensor to detect and track lanes and stop lines, and this information is used to measure the lateral and longitudinal distances.

An EKF-based GPS/DR error estimation filter estimates the GPS/DR error using lateral/longitudinal distance measurements that are obtained from the image processing system. In this study, we have assumed that the waypoint (map) is very accurate, and it makes GPS/DR error estimation possible. If the waypoint has an error, then the error is considered as a GPS/DR position error. Thus the positioning result of the proposed system is not sufficiently accurate in a global frame (erroneous latitude and longitude) but is accurate in a waypoint frame to make safe autonomous driving possible.

### 4.1. GPS/DR Error Modeling

In an open sky, the error components can be modeled by using the Dilution of Precision (DOP) error component that results of the placement of the satellites with the exception of the thermal noise, clock errors and the atmosphere error components [19,20]. The range measurements that can be obtained from a satellite can be modeled as follows:

(4) $$\rho_{i}=p+dp+c\left(dt-dT\right)+d_{atmosphere}+\varepsilon_{\rho }$$

**Figure 3:**
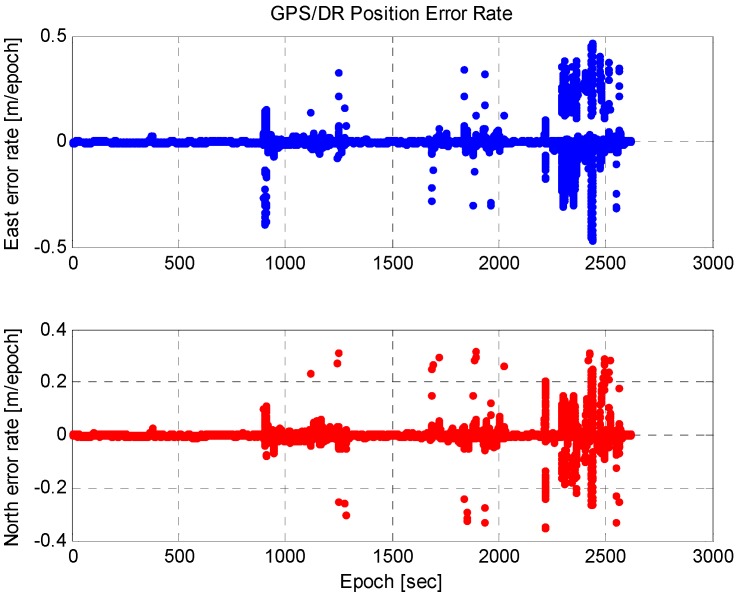
GPS/DR position error rate.

In this equation, $\rho_{i}$ is the range measurement of a satellite i, dp, dt, dT, $d_{atmosphere}$, $\varepsilon_{\rho }$, represent the geometrical distance between a satellite and a receiver, the orbit error of a satellite, the clock error of a satellite, the clock error of the receiver, the atmospheric error (the delay error due to the ionosphere and troposphere), and the thermal noise of the receiver, respectively. At least four range measurements are required to calculate the position, and non-lane areas are very short during autonomous driving. At an intersection, the length of the section is shorter than 100 m and can be driven within 10 s at a speed of 36 km/h. We can assume that there is no change in the DOP during short intervals, and therefore, GPS errors can be modeled as a random constant. As shown in Figure 3, the random constant model is appropriate because the change in the GPS/DR position error is small. We correct the GPS/DR position with the estimated GPS error by using the waypoint and vision in the lane or by predicting the GPS/DR error in the non-lane [21]:

(5) $$x_{k}=\left[\begin{array}{c} e_{E}\\ e_{N} \end{array}\right],x_{k+1}=\left[\begin{array}{cc} 1 & 0\\ 0 & 1 \end{array}\right]\left[\begin{array}{c} e_{E}\\ e_{N} \end{array}\right]$$

$e_{\left(\cdot \right)}$ shows the east and north GPS/DR errors as state variables.

### 4.2. Curve Model Lane Detection, Curved Parameter Estimation and Lateral Measurement

**Figure 4:**
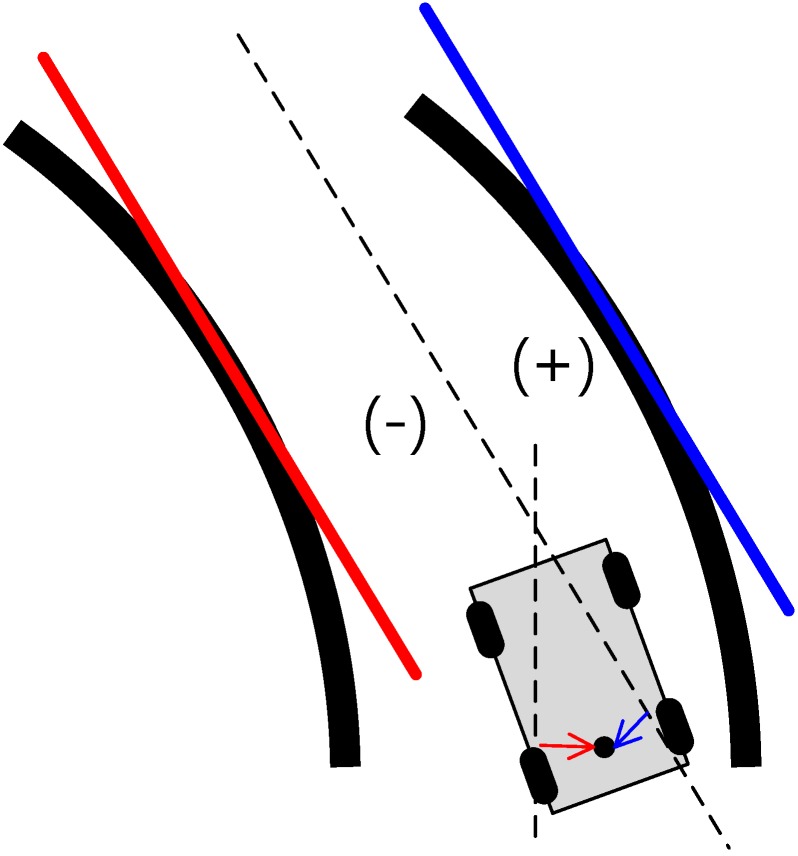
Lateral error during lane detection with a straight line model.

In general, the lane detection methods trace the vanishing points by using a straight line model [22]. When the lane detection methods use a straight line model, an error can occur when lateral measurements are made in sections with a curved line (Figure 4).

**Figure 5:**
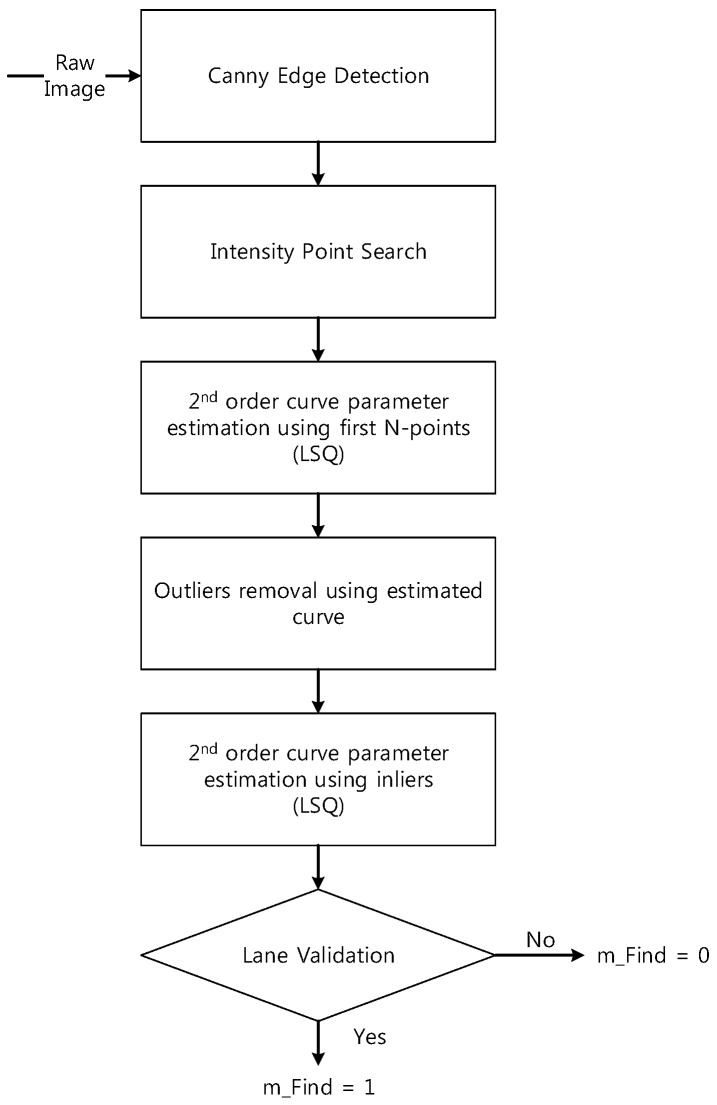
Flowchart for the lane detection in a curved model.

**Figure 6:**
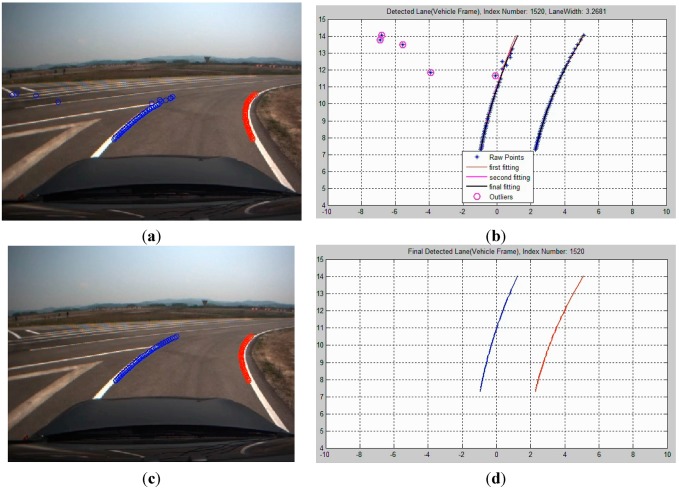
Removal of the outliers through curved model estimation: (**a**) image frame; (**b**) vehicle frame; (**c**) before the outlier removal; (**d**) after the outlier removal.

Figure 4 shows that the origin of the vehicle is on the right side of the lane (+), but errors occur in the estimation of the left side of the lane (−) during lane detection using straight line models. Therefore, lane detection is carried out using curved models to remedy these errors. Figure 5 consists of a flow chart of the lane detection with curved models. The curved parameters of the lane are estimated by generating a model with a second-order polynomial line by using the least squares (LSQ) method with a simple edge detector, finding pixels that are considered to indicate lanes, and using *n* points among them. Subsequently, an outlier is removed by using the estimated line for the curve, as in Figure 6.

The constraints of the lane validation method used in this paper are as follows:

(1) The width of both lanes that are detected is 2.5–4.5 m. The width of the general lane is about 3.5 m.
(2) The difference in the slopes (the first-order term) for both lanes is less than 0.3 because the lanes are parallel.
(3) The difference in the angle variation (the second-order term) for both lanes is below 0.015 because the variation in the angle of both lanes is the same as that in curved sections.

The length of the area is confined to be 14 m in order to minimize the distortion that occurs during transformation from an image frame to a vehicle frame, and M=50, n=30 are also set.

To obtain stable lateral measurements, the curved parameter ($x_{l,k}$) is estimated by using a Kalman filter with the points predicted for the outlier-removed lanes and the curved models consist of the second-order polynomial models from Equation (6):

(6) $$y\left(x\right)=a+bx+cx^{2}$$

This simple model is used with vehicles that move at a speed of *ν*, as measured by the odometer of the vehicle, and the filter equation is as follow with Δs=v⋅Δt [23,24].

State equation:

(7) $$F_{l,k-1}=\left[\begin{array}{ccc} 1 & \Delta s & \frac{\Delta s^{2}}{2}\\ 0 & 1 & \Delta s\\ 0 & 0 & 1 \end{array}\right],{\hat{x}}_{l,k}^{\left(-\right)}=F_{l,k-1}{\hat{x}}_{l,k-1}^{\left(+\right)}+W_{l,k-1}$$

(8) $$P_{l,k}^{\left(-\right)}=P_{l,k-1}^{\left(+\right)}\cdot F_{l,k-1}\cdot P_{l,k-1}^{\left(+\right)}+Q_{l,k-1}$$

Measurement equation:

(9) $$z_{l,k}=\frac{P_{vehicle,left,X}+P_{vehicle,right,X}}{2}$$

(10) $$H_{l,k}=\left[\begin{array}{ccc} 1 & P_{vehicle,Y} & {P_{vehicle,Y}}^{2}\\ \vdots & \vdots & \vdots \\ 1 & P_{vehicle,Y} & {P_{vehicle,Y}}^{2} \end{array}\right]$$

(11) $$K_{l,k}=P_{l,k}^{\left(-\right)}\cdot H_{l,k}^{\text{T}}{\left[H_{l,k}\cdot P_{l,k}^{\left(-\right)}\cdot H_{l,k}^{\text{T}}+R_{l,k}\right]}^{-1}$$

(12) $${\hat{x}}_{l,k}^{\left(+\right)}={\hat{x}}_{l,k}^{\left(-\right)}+K_{l,k}\left(z_{l,k}-H_{l,k}\cdot {\hat{x}}_{l,k}^{\left(-\right)}\right)$$

(13) $$P_{l,k}^{\left(+\right)}=\left(I-K_{l,k}H_{l,k}\right)P_{l,k}^{\left(-\right)}$$

**Figure 7:**
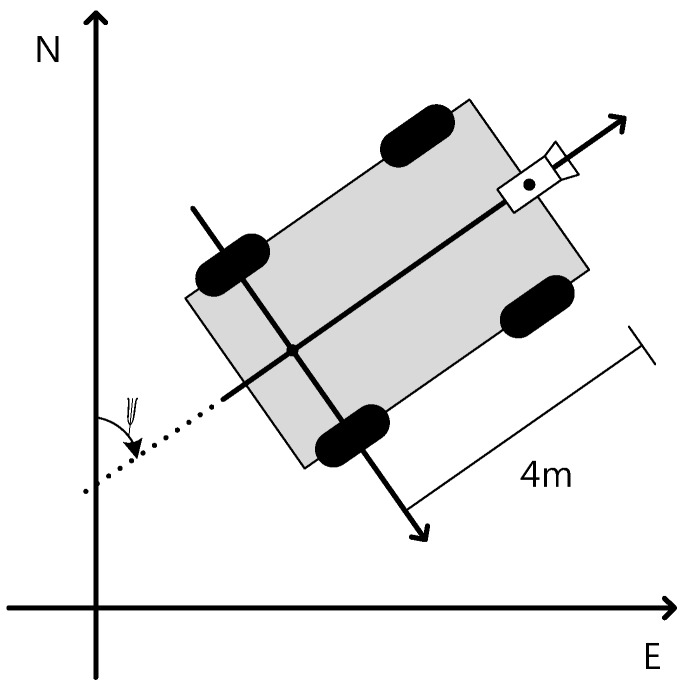
Global frame and vehicle frame.

The frame is the vehicle frame (Figure 7) with $P_{vehicle}$ detected lane points in the vehicle frame. The points detected on the Y axis for both lanes is estimated using a LSQ method that corresponds to $P_{vehicle,left,Y}=P_{vehicle,right,Y}=P_{vehicle,Y}$.

**Figure 8:**
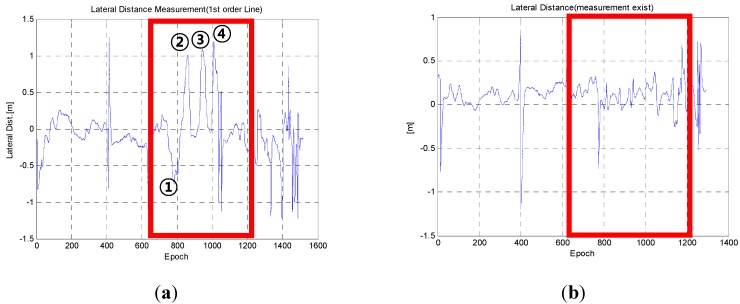
Lateral measurements for lane detection: (**a**) in a straight line model; (**b**) in the curved model.

**Figure 9:**
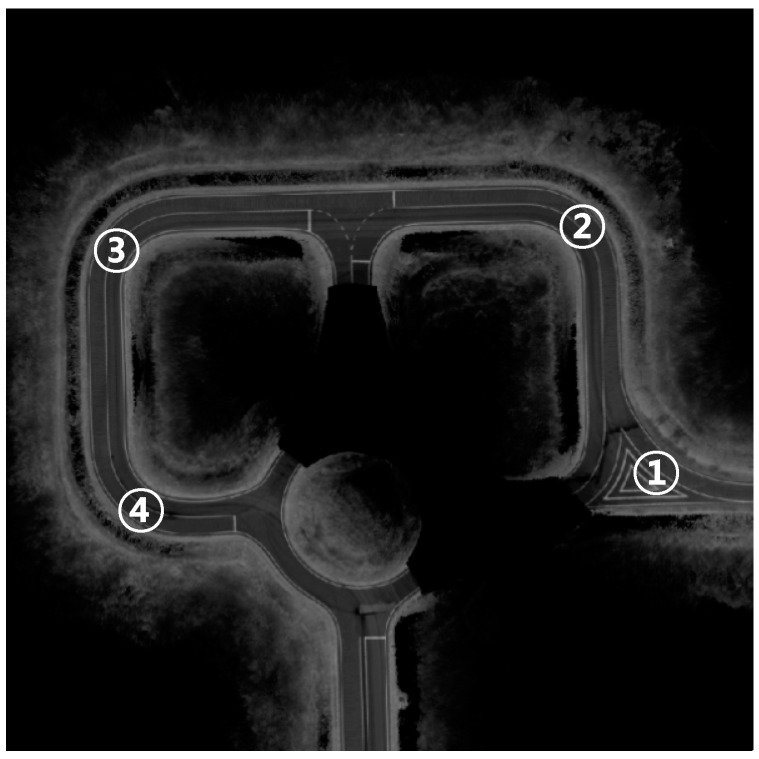
Curved lane sections.

Figure 8 represents the lateral measurements for all sections and the section that is indicated has a sharp curved lane that appears sequentially (Figure 9).

The results were obtained using driving data that was obtained from the center of the lanes with manual driving. Therefore, the lateral measurements should have a value near zero. In Figure 9, area ① is a right turn, and ②–④ are a left turn. As shown in Figure 8a, the errors indicate that the vehicles are located in the left of the lane in the left turn area and that vehicles are located on the right side of the lane in the right turn area. As the curved lane sections become sharper, the errors increase. If this measurement is used, the GPS/DR errors can be incorrectly estimated. However, Figure 8b shows lateral measurements close to zero with the second-order polynomial models. In other words, the estimation functions for GPS/DR errors can be improved by using curved models.

### 4.3. Longitudinal Measurements from the Stop Line Detection

**Figure 10:**
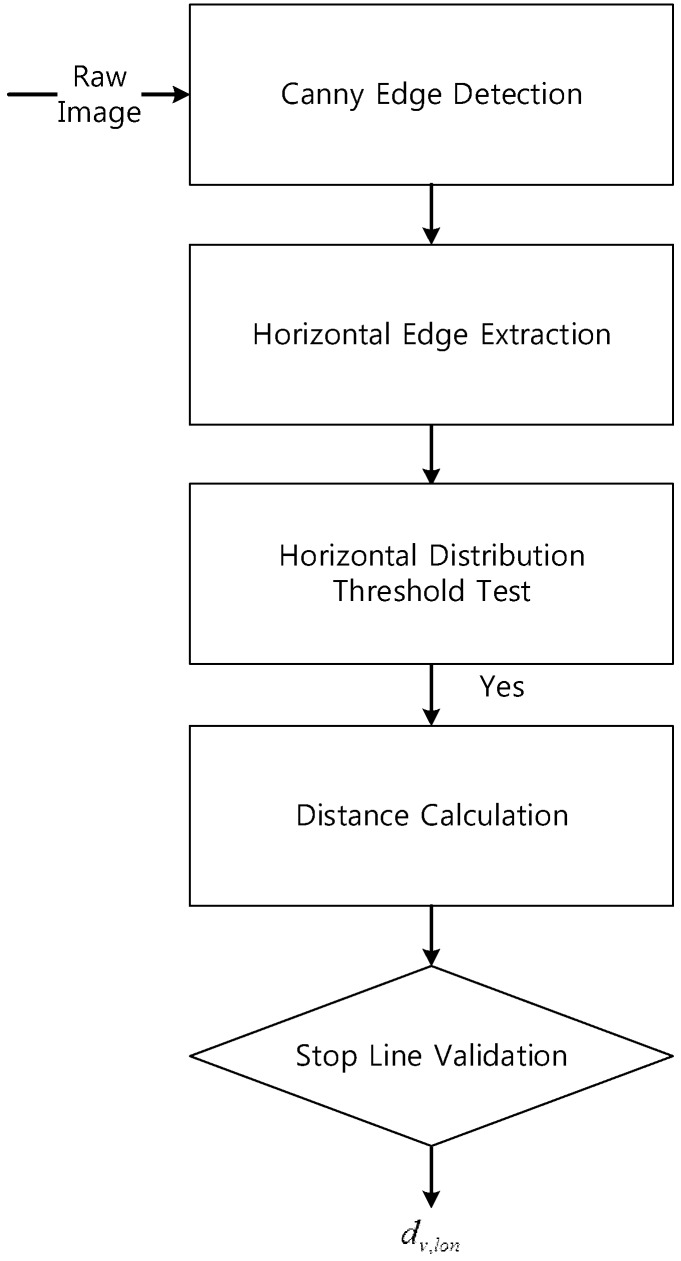
Flowchart for the stop line detection.

**Figure 11:**
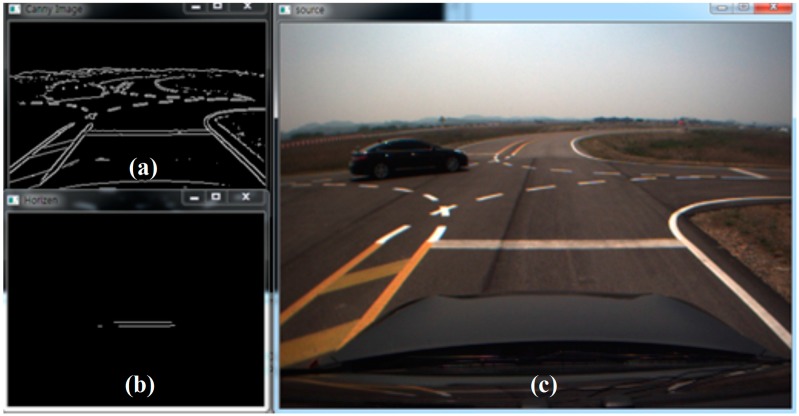
Result of the stop line detection: (**a**) Canny edge detection; (**b**) detected stop line; (**c**) raw image.

An accurate position in terms of the lateral lanes is used to keep the vehicle in the center of the lane. However, the accuracy of the longitudinal position is important in order to enact changes in the direction when in an intersection. Stop lines can be detected using vision sensors, and the stop lines that are detected are thus used to calculate the longitudinal measurements. Figure 10 shows the flowchart of stop line detection and the result is shown as Figure 11. The constraints of the validation of the distance for the detected stop lines are as follows:

(1) The stop line is located laterally within 3.5 m.
(2) The stop line measurement is less than 14 m (which is the reliable range of vision calibration in this test).
(3) The difference between the detected stop line location and the map is less than 3 m (feasible GPS/DR error).

### 4.4. GPS/DR Error Estimation Filter

The error estimation filter uses measurements from both sensors, including GPS/DR and a vision sensor. GPS/DR provides information on the absolute position, and the vision sensor provides information on the vehicle frame that is different from that of GPS/DR. Therefore, the error estimation filter has a structure where the information of the navigation frame is corrected by using information that is measured for the vehicle frame [25]. The lateral distance between the GPS/DR and the waypoint link can be calculated by using a waypoint as the information of a map. For the vision sensor, the lateral distance from the center of the lane can be measured by detecting the lanes. This study makes two assumptions for the error estimation filter:

*Assumption 1*:  The waypoint is located at the center of the lanes. In general, the location information for the lanes is produced through a survey when accurate maps are produced, and therefore, the center of the lanes can be easily extracted from an accurate map that has been produced.

*Assumption 2*:  The waypoints in the curved section are very fine, so the vehicles do not go outside of the lane.

The measurement that was used for the error estimation filter utilizes the lateral distance from the center of lanes that was measured with the vision sensor ($d_{v,lat}$), and the lateral distance is calculated by using the GPS/DR and the waypoint ($d_{g,lat}$). The longitudinal distance uses $d_{v,lon}$ and $d_{g,lon}$, which are obtained from the vision sensor and from GPS/DR, respectively. Therefore, the measurement equation is shown in Equation (14):

(14) $$z_{e,k}=\Delta d_{k}=\left[\begin{array}{c} d_{v,lat,k}-d_{g,lat,k}\\ d_{v,lon,k}-d_{g,lon,k} \end{array}\right]$$

**Figure 12:**
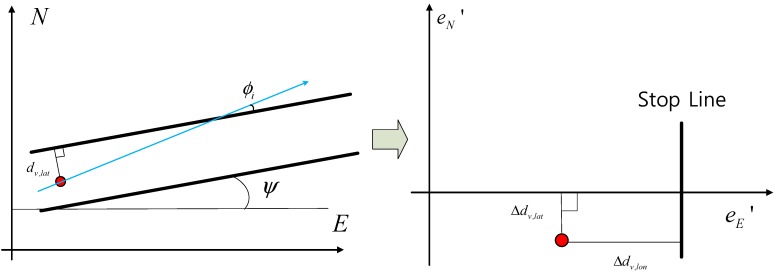
Lane frame.

It is necessary to present the lane frame to configure the filter (Figure 12), and as shown in Figure 12, the frame is rotated according to the heading angle (−ψ) and is calculated from a waypoint. Then, it can be transformed into a lane frame:

(15) $$R\left(-\psi_{k}\right)=\left[\begin{array}{cc} \cos\psi_{k} & \sin\psi_{k}\\ -\sin\psi_{k} & \cos\psi_{k} \end{array}\right]$$

After the rotation, the transformed ${e_{\left(E\right)}}^{\prime }$ axis corresponds to a lane. This is the lateral distance information of the lanes, and therefore, the equation for the lane frame can be presented as follows:

(16) $$-e_{N}\prime =-\Delta d_{v,lat,k}=-e_{E}\cdot \sin\psi_{k}+e_{N}\cdot \cos\psi_{k}$$

(17) $$e_{E}\prime =\Delta d_{v,lon,k}=e_{E}\cdot \cos\psi_{k}+e_{N}\cdot \sin\psi_{k}$$

(18) $$h_{e,k}(x)=\left[\begin{array}{c} \Delta d_{v,lon,k}\\ \Delta d_{v,lat,k} \end{array}\right]=\left[\begin{array}{c} e_{E}\cdot \cos\psi_{k}+e_{N}\cdot \sin\psi_{k}\\ e_{E}\cdot \sin\psi_{k}-e_{N}\cdot \cos\psi_{k} \end{array}\right]$$

Equation (19) can be obtained through a partial differential of each term in Equation (18):

(19) $$H_{e,k}\left(x\right)=\left[\begin{array}{cc} \frac{h_{e,k}\left(x\right)}{\partial E} & \frac{h_{e,k}\left(x\right)}{\partial N} \end{array}\right]=\left[\begin{array}{cc} \cos\psi_{k} & \sin\psi_{k}\\ \sin\psi_{k} & -\cos\psi_{k} \end{array}\right]$$

The GPS/DR error estimation filter can be summarized as follows:

State equation:

(20) $$x_{e}=\left[\begin{array}{c} e_{E}\\ e_{N} \end{array}\right],F_{e,k-1}=\left[\begin{array}{cc} 1 & 0\\ 0 & 1 \end{array}\right],x_{e,k}=F_{e,k-1}\cdot x_{e,k-1}+W_{e,k-1}$$

(21) $$P_{e,k}^{\left(-\right)}=P_{e,k-1}^{\left(+\right)}\cdot F_{e,k-1}\cdot P_{e,k-1}^{\left(+\right)}+Q_{e,k-1}$$

Measurement equation:

(22) $$z_{e,k}=\Delta d_{k}=\left[\begin{array}{c} d_{v,lat,k}-d_{g,lat,k}\\ d_{v,lon,k}-d_{g,lon,k} \end{array}\right]$$

(23) $$H_{e,k}\left(x\right)=\left[\begin{array}{cc} \cos\psi_{k} & \sin\psi_{k}\\ \sin\psi_{k} & -\cos\psi_{k} \end{array}\right]$$

(24) $$K_{e,k}=P_{e,k}^{\left(-\right)}\cdot H_{e,k}^{\text{T}}{\left[H_{e,k}\cdot P_{e,k}^{\left(-\right)}\cdot H_{e,k}^{\text{T}}+R_{e,k}\right]}^{-1}$$

(25) $${\hat{x}}_{e,k}^{\left(+\right)}={\hat{x}}_{e,k}^{\left(-\right)}+K_{e,k}\left(z_{e,k}-H_{e,k}\cdot {\hat{x}}_{e,k}^{\left(-\right)}\right)$$

(26) $$P_{e,k}^{\left(+\right)}=\left(I-K_{e,k}H_{e,k}\right)P_{e,k}^{\left(-\right)}$$

### 4.5. Longitudinal Measurement from the Curve Matching

The longitudinal position error from the GPS/DR measurement may be large in the transition area from a straight to a curved lane when the vehicle starts to drive or drives in one direction for a long time because there is no longitudinal information. If the vehicle enters a curved section, the heading of the vehicle and the waypoint change, and then the error estimation for east and north become available. However, before entering a curve, the waypoint navigation may suffer from a failure due to the steering command that has been generated with an incorrect timing as a result of the longitudinal error.

The curved parameter of the lane ahead can be calculated as in Equation (6) by using the waypoint. After interpolation with a resolution of Δy, the waypoint that uses the curved parameter that has been calculated is transformed into a vehicle frame. At this time, the transformation uses the corrected position with the error estimator that has been laterally corrected. Then the interpolated waypoint curve ($l_{wp}^{v}$) has no lateral distance with the curve ($l_{i}^{v}$) that is detected from the image and only has a longitudinal distance because the lateral error of the GPS/DR has already been estimated. The error function that measures the longitudinal range is shown in Equations (27) and (28):

(27) $$e=l_{wp}^{v}\left(x\right)-l_{i}^{w}\left(x\right)$$

(28) $$d_{v,lon}=\arg\min\sum e^{2}$$

**Figure 13:**
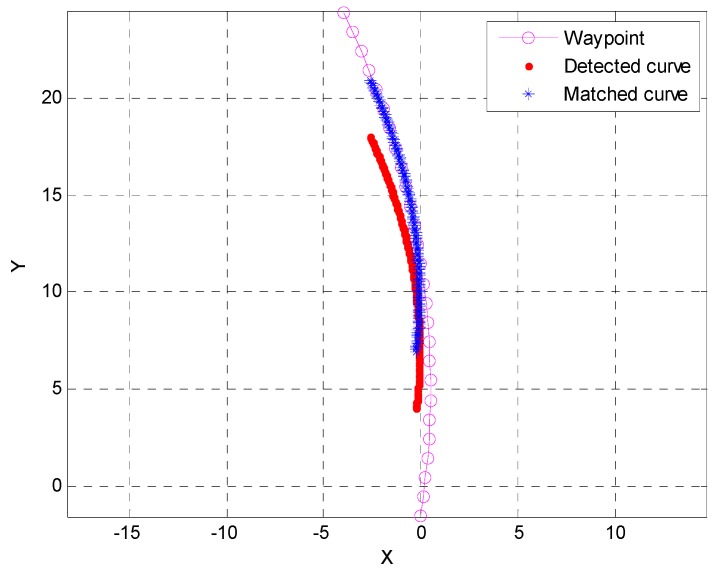
Measuring the longitudinal distance with curve matching (o: waypoint curve; ●: detected curve lane, *: matched curve).

As shown in Figure 13, the true longitudinal distance is −2.78 m, and the distance measured ($d_{v,lon}$) from Equation (28) is −2.93 m. Thus, accurate longitudinal measurements can be obtained through curve matching.

The measurements for the longitudinal range from the curve matching are effective only in the transition from a straight to a curved lane. After the longitudinal error has been estimated once, the lateral information in the area of the curve becomes longitudinal information. Therefore, in this study, we have used Δy=0.1 m and have restricted the section where curve matching was performed by the waypoint curvature. The longitudinal range that was measured is used as an input of the error estimation filter that is described in Section 4.4.

## 5. Autonomous Experimental Results

**Figure 14:**
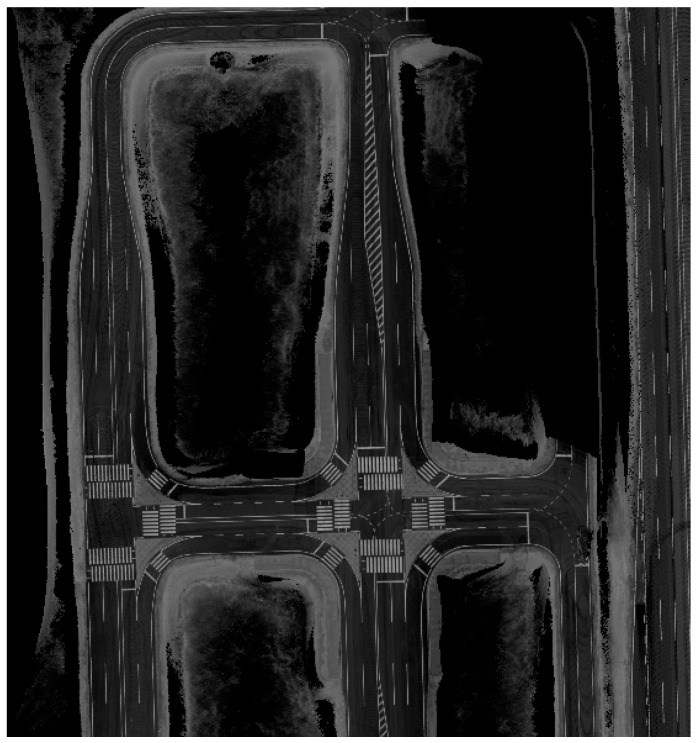
Reflectivity map.

The experiment was carried out by applying the lane detection method with a curved model and the GPS/DR error estimation filter proposed in this study in an autonomous vehicle. Figure 14 shows a map that is based on the reflectivity of the experimental site. The map was produced by using a commercial RTK/INS system and a 3D-LiDAR (HDL-32E, Velodyne, Morgan Hill, CA, USA) (Table 1).

**Table 1:** Experimental environment.

GPS/DR	Vision Sensor	Reference Trajectory
U-Blox EVK 6T + ADIS 16405	BumbleBee 2	Commercial RTK/INS (Novatel Propak V3 + SPAN HG1700)

**Figure 15:**
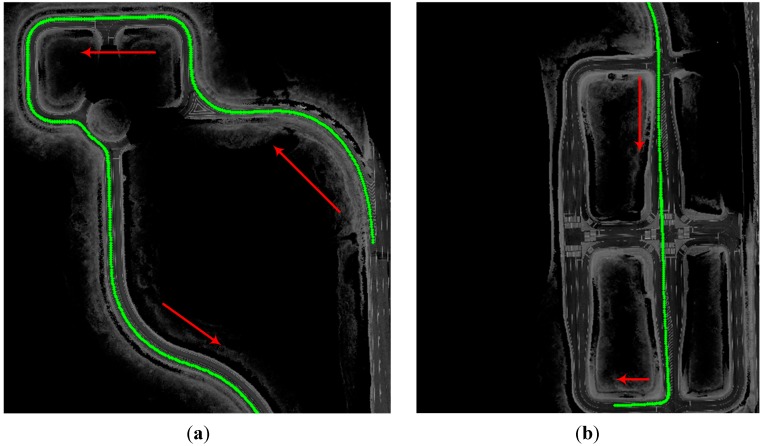
Driving trajectory (arrows represent the direction of vehicle). (**a**) curved section; (**b**) intersection.

The true value of the position cannot be known due to the movement in the environment, so a commercial RTK/INS system, which has a high price, was used to determine the reference position (true value) to conduct a quantitative analysis. Figure 15 shows the total trajectory.

**Figure 16:**
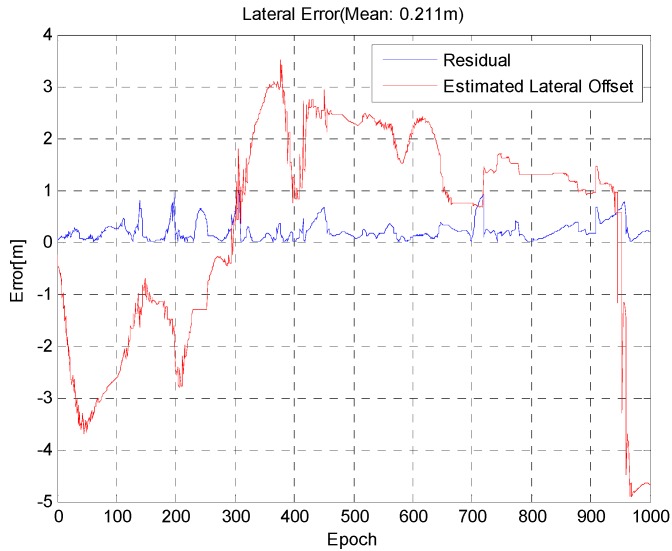
Estimated GPS/DR lateral error (red) and position error (blue).

**Figure 17:**
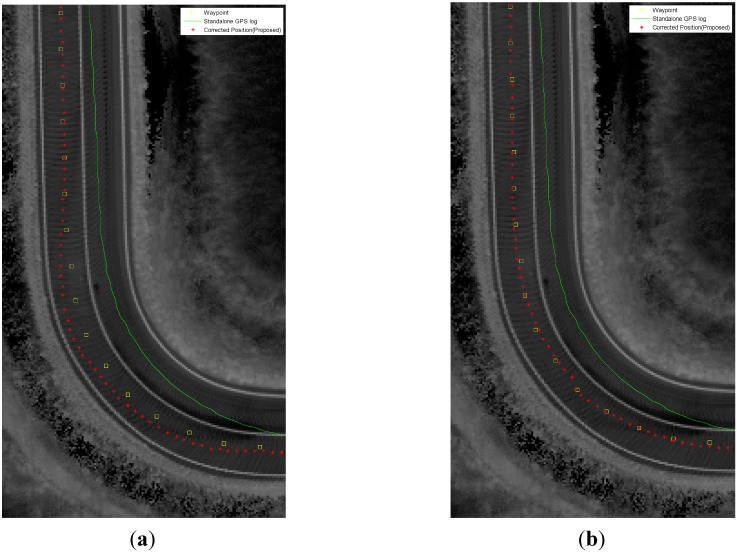
Experimental results for the curved lane sections: (**a**) straight model; (**b**) curved model (□: waypoint, -: Standalone GPS/DR, *: proposed).

Figure 16 shows the results that were obtained with the proposed method, and Figure 17 shows a comparison of the results when measurements were used for lane detection with a straight line model and with a curved model in sections with sharp curves. In this section, the result of using a straight model is that vehicles lean to the left during a right turn and to the right during a left turn. However, the results when using a curved model reduce the lateral error.

**Figure 18:**
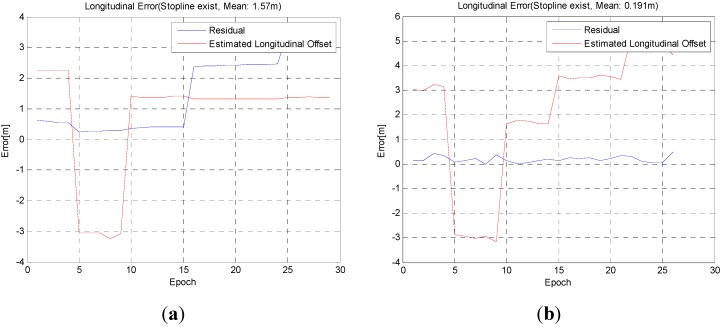
Estimated GPS/DR longitudinal error (red) and position error (blue): (**a**) without stop line detection; (**b**) with stop line detection.

A stop line does not exist in every section, and therefore, the longitudinal accuracy analyzes the sections with stop lines (Figure 18).

**Figure 19:**
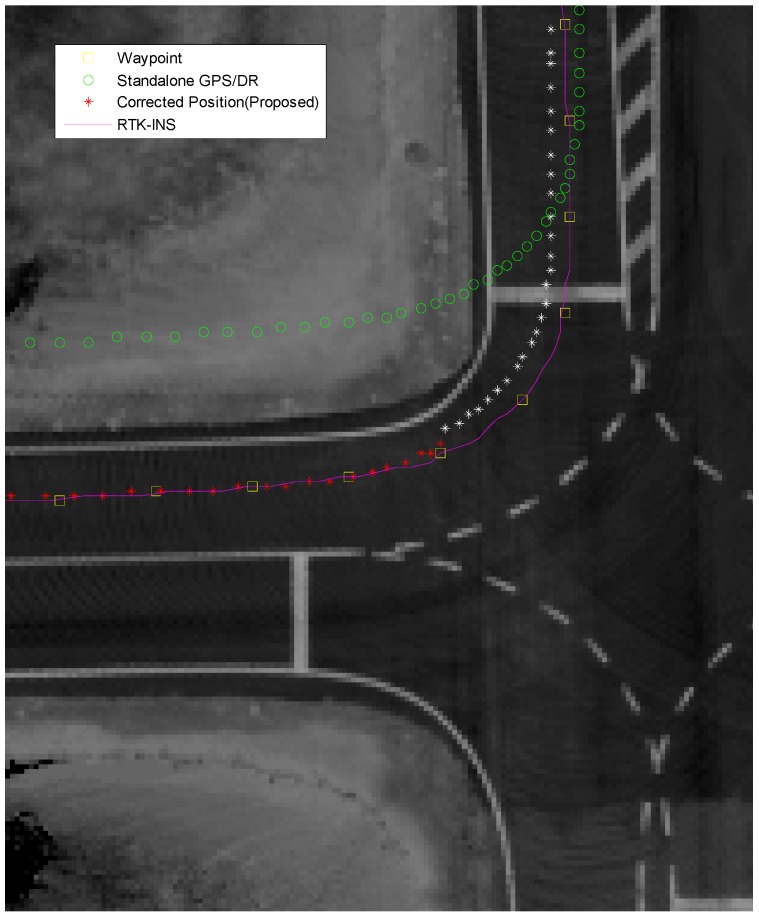
Result at the intersection (□: waypoint, o: GPS/DR, *: proposed, -: RTK/INS).

**Figure 20:**
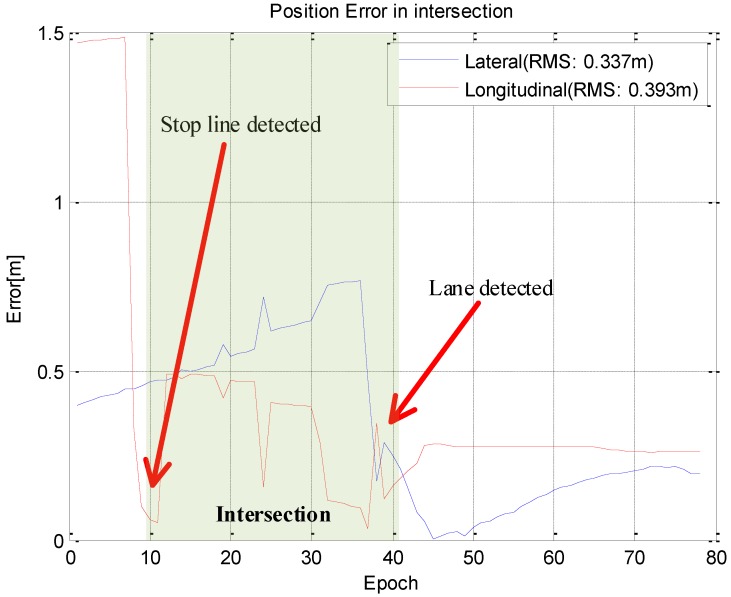
Position error at the intersection.

Figure 19 and Figure 20 show the results in an intersection and the white asterisk indicates the results where no vision measurements have been taken. Even in this section, vision information cannot be obtained, we derived the position with a sub-meter level of accuracy.

In Table 2, the overall longitudinal error is larger than the intersection. The reason for this is that the longitudinal error can be corrected before entering an intersection, but cannot be estimated because most of total trajectory is straight.

**Table 2:** Result summary.

RMSE (m)	GPS/DR	Overall	w/o Stop Line	With Stop Line	Intersection
Lateral	1.77	0.217	-	-	0.337
Longitudinal	2.11	0.618	1.57	0.191	0.393

## 6. Conclusions

This study proposes a navigation system that supports autonomous driving through the use of GPS/DR, waypoints and a vision sensor. The disadvantages of using a lane following system with lane detection is that there are discontinuous sections in the lanes. Therefore, this study proposed a GPS/DR error estimation filter that allows stable navigation in such sections. In addition, we suggest methods that can reduce the errors in the lateral distance by detecting lanes using a curved model in order to solve the problems where lateral distance errors occur in curved lane sections. The curve matching method between the waypoints and the detected curve lanes reduces the longitudinal error before entering a curved section after a straight section. Only lane and stopline detection was performed using image processing, so much less computational power is required when compared to the use of visual odometry and a particle filter, and it is thus very easy to enable real-time use in embedded systems.

This system can therefore be applied in not only an autonomous vehicle but also vehicles which is already equipped with GPS, IMU and black box(monocular vision sensor) to safely run the vehicle in straight lanes, curved lanes and through intersections.

However, greater availability is required for autonomous vehicles. The stopline was the only road marking considered in this paper for longitudinal information, but the stopline does not exist everywhere. Thus, every road marking (arrow, speed limit, *etc.*) will be added to provide more longitudinal information. Also, vision sensors have limited functionality at night or with a backlight, so LiDAR, Dedicated Short Range Communications (DSRC), *etc.* can possibly be used in autonomous vehicles [26,27,28,29]. Multi-sensor systems based on a federated Kalman filter can improve the reliability and availability of the localization system, and in addition, the integrity of an autonomous vehicle can be provided by using the covariance of local filters and the master filter. Therefore, a localization method that ensures the availability and integrity will be part of future work.

## References

[1] Lee B.H., Jee G.I. Performance analysis of GPS-RTK floating solution with Doppler measurement. Proceedings of the IS-GPS/GNSS.

[2] Serrano L., Kim D., Langley R.B. A single GPS receiver as a real-time, accurate velocity and acceleration sensor. Proceedings of the ION GNSS 17th ITM.

[3] Badino H., Huber D., Kanade T. Visual topometric localization. Proceedings of the IEEE Intelligent Vehicles Symposium.

[4] Du J., Barth M.J. (2008). Next-Generation Automated Vehicle Location Systems: Positioning at the Lane Level. IEEE Trans. Intell. Transp. Syst..

[5] Laneurit J., Chapuis R., Chausse F. (2005). Accurate vehicle positioning on a numerical map. Int. J. Control Autom. Syst..

[6] Miller I., Campbel M., Huttenlocher D. (2011). Map-aided localization in sparse global positioning system environments using vision and particle filtering. J. Field Robot..

[7] Dissanayake M.W.M.G., Newman P., Clark S., Durrant-Whyte H.F., Csorba M. (2001). A solution to the simultaneous localization and map building (SLAM) problem. IEEE Trans. Robot. Autom..

[8] Montemerlo M., Thrun S., Koller D., Wegbreit B. FastSLAM: A factored solution to the simultaneous localization and mapping problem. Proceedings of the AAAI National Conference on Artificial Intelligence.

[9] Thrun S., Burgard W., Fox D. (2005). Probabilistic Robotics.

[10] Levinson J., Montemerlo M., Thrun S. Map-based precision vehicle localization in urban environments. Proceedings of the Robotics: Science and Systems.

[11] Levinson J., Thrun S. Robust vehicle localization in urban environment using Probabilistic Maps. Proceedings of the IEEE International Conference on Robotics and Automation.

[12] Schreiber M., Knoppel C., Franke U. LaneLoc: Lane marking based localization using highly accurate maps. Proceedings of the IEEE Intelligent Vehicles Symposium.

[13] Bak A., Gruyer D., Bouchafa S., Aubert D. Multi-sensor localization—Visual odometry as a low cost proprioceptive sensor. Proceedings of the 15th International IEEE Conference on Intelligent Transportation Systems.

[14] Cuong N.V., Heo M.B., Jee G.I. (2013). 1-Point Ransac based robust visual odometry. J. Korean GNSS Soc..

[15] Scaramuzza D., Siegwart R. (2008). Appearance-guided monocular omnidirectional visual odometry for outdoor ground vehicles. IEEE Trans. Robot..

[16] Gruyer D., Belaroussi R., Revilloud M. Map-Aided localization with lateral perception. Proceedings of the IEEE Intelligent Vehicles Symposium.

[17] Ieng S.S., Gruyer D. Merging lateral cameras information with proprioceptive sensors in vehicle location gives centimetric precision. Proceedings of the 18th International Technical Conference on the Enhanced Safety of Vehicles (ESV).

[18] Li H., Nashashibi F., Toulminet G. Localization for intelligent vehicle by fusing mono-camera, low-cost GPS and map data. Proceedings of the International IEEE Annual Conference on Intelligent Transportation Systems.

[19] Bernhard H.-W., Herbert L., Elma W. (2008). GNSS-Global Navigation Satellite Systems, GPS, GLONASS, Galileo & More.

[20] Kaplan E.D., Hegarty C.J. (2005). Understanding GPS: Principles and Applications.

[21] Seo S.H., Lee B.H., Jee G.I. Position error correction using waypoint and vision sensor. Proceedings of the International Symposium on GNSS.

[22] Kuk J.G., An J.H., Ki H.Y., Cho N.I. Fast lane detection & tracking based on hough transform with reduced memory requirement. Proceedings of the International IEEE Annual Conference on Intelligent Transportation Systems.

[23] Li T., Zhidong D. A new 3D LIDAR-based lane markings recognition approach. Proceedings of the IEEE International Conference on Robotics and Biomimetics.

[24] Bevly D.M. (2010). GNSS for Vehicle Control.

[25] Lee B.H., Im S.H., Heo M.B., Jee G.I. Error correction method with Precise Map Data for GPS/DR based on Vision/Vehicle Speed Sensor. Proceedings of the ION GNSS+.

[26] Alam N., Balaei A.T., Dempster A.G. (2012). An instantaneous Lane-Level positioning using DRSC carrier frequency offset. IEEE Trans. Intell. Transp. Syst..

[27] Chen L., Li Q., Li M., Zhang L., Mao Q. (2012). Design of a multi-sensor cooperation travel environment perception system for autonomous vehicle. Sensors.

[28] Chu T., Guo N., Backen S., Akos D. (2012). Monocular Camera/IMU/GNSS integration for ground vehicle navigation in challenging gnss environments. Sensors.

[29] Cong L., Li E., Qin H., Ling K.V., Xue R. (2015). A performance improvement method for low-cost land vehicle GPS/MEMS-INS attitude determination. Sensors.

